# Risk Factors and Common Organisms in Bacterial Keratitis in a Tertiary Center in Iran

**DOI:** 10.18502/jovr.v21.18405

**Published:** 2026-07-15

**Authors:** Yasaman Hadi, Saba Gholamalizadeh, Leila Ghiasian, Negar Dadpour, Navid Elmi Sadr, Marjan Mazouchi

**Affiliations:** ^1^Eye Research Center, Five Senses Institute, Rassoul Akram Hospital, Iran University of Medical Sciences, Tehran 1634674651, Iran; ^2^Department of Ophthalmology, School of Medicine, Semnan University of Medical Sciences, Semnan, Iran; ^3^Bina Eye Hospital, Tehran, Iran; ^4^Ophthalmic Research Center, Research Institute for Ophthalmology and Vision Science, Shahid Beheshti University of Medical Sciences, Tehran, Iran; ^5^The preprint version of this study has been available online. https://doi.org/10.21203/rs.3.rs-4799498/v1. This work is licensed under a CC BY 4.0 License.

**Keywords:** Corneal Ulcer, Keratitis, Ocular Trauma, Risk Factors, Surgery

## Abstract

**Purpose:**

This study aimed to document the spectrum of isolated microorganisms and investigate the risk factors associated with infectious keratitis over a 12-year period at a tertiary referral center.

**Methods:**

A retrospective analysis was conducted on the medical records of 867 patients diagnosed with infectious keratitis between 2009 and 2021. Corneal scraping was performed for 414 patients, and all individuals received fortified empiric antibiotic eye drops. Surgical intervention was conducted for 376 patients during their hospital stay.

**Results:**

Trauma emerged as the most prevalent risk factor (21.9%). The main causative microorganism for corneal ulcers was found to be *Pseudomonas aeruginosa*. *Staphylococcus aureus* was the primary microorganism isolated in patients below 50 years old, whereas *P. aeruginosa* was more commonly identified in patients aged 50 and above. Tarsorrhaphy was performed on 23.6% of the patients. Additionally, other surgical procedures were undertaken in 44.6% of the cases, with tectonic graft and corneal patch graft emerging as the most frequent surgical interventions.

**Conclusion:**

This study underscores the importance of trauma as the leading predisposing factor and *P. aeruginosa* as the predominant microorganism isolated in cases of adult microbial corneal ulcers in Iran.

##  INTRODUCTION

Corneal ulcers, characterized by damage to the corneal epithelium and subsequent infection of the underlying stroma, represent a serious ocular condition and a major cause of blindness in developing countries.^[1]^ Several risk factors may contribute to the development of corneal ulcers including mechanical trauma from wooden materials, chemical injuries, and contact lens use.^[2]^ The causative pathogens may include bacteria, viruses, and *Acanthamoeba*, each capable of leading to severe visual impairment if left untreated.^[3]^

Early recognition and treatment of microbial keratitis is essential to prevent corneal clouding, scarring, or perforation. Accurate diagnosis and timely selection of appropriate antibiotics play a central role in visual outcomes.^[4]^ Empirical therapy should often be initiated based on the organisms most prevalent in a given geographic region rather than waiting for definitive culture results to minimize delays in treatment.^[4]^

The global incidence of corneal ulcers varies significantly. In the USA, the annual incidence is estimated at approximately 28 cases per 100,000 individuals.^[5]^ In contrast, in developing countries, the reported prevalence is much higher, reaching around 800 cases per 100,000 persons each year.^[6]^ This disparity underscores the influence of socioeconomic and healthcare-related factors on disease burden.

Several studies have explored the regional variation in causative organisms and risk factors for corneal ulcers. For example, a retrospective study by Soleimani et al at a referral eye hospital in Iran found that *Pseudomonas aeruginosa* was the predominant pathogen in younger individuals with bacterial keratitis, whereas *Streptococcus pneumoniae* was most commonly identified in patients over 50 years old.^[7]^ Notably, this study did not examine risk factors associated with corneal ulcers.^[7]^

The present study aims to address the knowledge gaps identified in prior research conducted in Iran, particularly concerning the risk factors and common microorganisms linked to bacterial keratitis within a tertiary care facility in Iran.

##  METHODS

This retrospective observational analysis was conducted at Rasoul Akram Hospital, a tertiary ophthalmology care center affiliated with Iran University of Medical Sciences in Tehran, Iran. The study focused on patients hospitalized for bacterial corneal ulcers between 2009 and 2021, and it was approved by the local ethics committee at Rasoul Akram Hospital (Approval number IR.IUMS.REC.1400.1087). Exclusions were made for patients with incomplete medical records, dendritic ulcers, and marginal or immune-mediated keratitis. Moreover, instances strongly suspected to involve fungal keratitis, those with positive microbiological assessments indicating a fungal cause, and cases where confocal scanning results pointed to potential fungal involvement were excluded.

In the medical records, we documented various factors including demographic characteristics, length of hospital stay, predisposing factors such as history of trauma, previous eye surgeries or conditions, and use of contact lenses. We also recorded smear and culture results, location, size, and depth of the ulcer, as well as the presence or absence of anterior chamber reaction and level of hypopyon. The location of the lesion was classified as central if it was within 5 mm of the corneal center, peripheral if it was within 3 mm of the limbus, and paracentral if it fell between these two locations. The size of the ulcer was determined by the largest diameter and categorized as small ( < 2 mm), medium (2-4 mm), or large ( > 4 mm).

All corneal scrapings were performed by a specialized cornea expert using a sterile surgical blade. The scrapings were obtained after the application of topical anesthesia. The procedures were conducted while closely examining the eye under slit lamp biomicroscopy. The corneal ulcer sample was divided onto two microscope slides for Gram and Giemsa staining. Fresh scalpel blades were then used to evenly spread the specimen onto three different culture media types (Sheep blood agar, chocolate agar, and Sabouraud dextrose agar). Subsequently, the plates were placed in an environment with 5% carbon dioxide at 37ºC for 96 hours. A positive culture was confirmed if distinct colonies of the same organism were observed on two solid media, or if there was continuous growth of the organism along the inoculation site on a single culture plate. The disk diffusion method was used to assess the antibiotic susceptibility of the microbial isolates. Although susceptibility testing offers important information, the retrospective nature of this study limits the data to the patients' documented medical histories. On admission, all patients received initial treatment with fortified empiric antibiotic eye drops containing vancomycin and ceftazidime. Subsequent adjustments to the antibiotic regimen were made based on the results obtained from culture testing.

The fortified drops were prepared using vancomycin (25-50 mg/mL), ceftazidime (50 mg/mL), amikacin (25 mg/mL equal to 2.5%), gentamicin (14 mg/mL equal to 0.3%), and cefazolin (50 mg/mL equal to 5%), all combined with preservative-free saline solution to achieve the desired final volume and concentration.

The treatment regimen was maintained until complete healing of the epithelial defect and resolution of stromal infiltration and anterior chamber inflammatory reaction were achieved. Topical corticosteroid eye drops were not administered during the treatment course to avoid exacerbation of bacterial infection.

Various surgical interventions were performed as needed, including conjunctival flap placement, blepharorrhaphy, application of tissue adhesive, amniotic membrane transplantation (AMT), patch grafting, tectonic penetrating keratoplasty, evisceration, and enucleation.

**Table 1:** The characteristics of corneal ulcers on admission

Risk factor (%)	
Blepharitis	125 (19.2)
Herpes	4 (0.6)
Trauma	143 (21.9)
Contact lens	52 (8)
Neurotrophic keratopathy	65 (10)
History of refractive surgery	42 (6.4)
History of other ocular surgeries	7 (1.0)
Dry eye disease	3 (0.5)
Other ophthalmic diseases	60 (9)
No risk factors	150 (23)
Location of ulcer (%)	
Central	342 (39.4)
Mid-peripheral	176 (20.3)
Peripheral	169 (19.5)
Total cornea	36 (4.2)
Not reported	144 (16.6)
Ulcer size (mm)	
Length	4.08 ± 2.00
Width	3.59 ± 1.99
Corneal stromal thinning (%)	309 (35.6)
Height of hypopyon (mm)	1.47 ± 1.07

**Table 2:** Outcomes of microbial culture pertaining to corneal ulcer

Positive culture (%)	
*Pseudomonas aeruginosa *	49 (28.2)
* Staphylococcus aureus*	42 (23.9)
* Staphylococcus epidermidis*	23 (12.8)
* Streptococcus pneumonia*	33 (18.8)
*Staphylococcus* coagulase negative	11 (5.9)
*Klebsiella*	7 (4.27)
*Streptococcus viridans*	3 (1.7)
*Escherichia coli*	3 (1.7)
*Enterobacteriaceae*	3 (1.7)
*Acinetobacter*	2 (0.8)

### Statistical Analysis 

Statistical analysis was conducted using SPSS version 23 (IBM Inc., Chicago, IL, USA). Descriptive statistics, including means and standard deviation, were used to analyze continuous variables, while categorical variables were presented in percentages. The prevalence of microbial isolates was compared using the chi-square test, and a logistic regression model was utilized to assess the risk factors. Statistical significance was considered for *P*-values < 0.05.

##  RESULTS

### Demographics

A total of 867 patients with unilateral bacterial keratitis were included. The cohort consisted of 502 males (57.6%) and 365 females (42.4%), with a mean age of 55.05 ± 24.25 years. The mean interval from symptom onset to hospital admission was 6.27 ± 5.77 days, and the average hospital stay was 7.44 ± 6.59 days.

### Risk Factors

Trauma was the most common predisposing factor (143 patients, 21.9%), followed by blepharitis (19.2%) and neurotrophic keratopathy (10%). Contact lens use was identified in 8% of cases. Refractive surgery history was present in 6.4% of patients. Surface ablation was the most common refractive surgery, and on average, patients experienced the onset of keratitis around 4.6 ± 1.2 days after the procedure. Notably, 150 patients (17.3%) had multiple identifiable risk factors, and 150 patients (23%) had no documented risk factor [Table 1].

### Corneal Ulcer Characteristics

Ulcer location was central in 342 (39.4%), mid-peripheral in 176 (20.3%), and peripheral in 169 (19.5%) cases. Total corneal involvement was present in 36 cases (4.2%). In 144 patients (16.6%), location data were missing. On average, the ulcers measured 4.08 ± 2.00 mm in length and 3.59 ± 1.99 mm in width. Corneal stromal thinning was observed in 309 cases (35.6%), and hypopyon was present with a mean height of 1.47 ± 1.07 mm. A total of 123 (14%) patients were diagnosed with a perforated corneal ulcer leading to a flat or shallow anterior chamber. Table 1 displays the risk factors associated with ulcers, the location, length, and width of the ulcers on the cornea, the presence of corneal thinning, and the height of hypopyon on admission.

### Culture Results

Of the 867 patients, 414 (47.7%) underwent microbial culture. Positive culture results were obtained in 176 cases (42.5%), while 238 (57.5%) showed no growth. The most common isolate was *P. aeruginosa* (28.2%), followed by *S. aureus* (23.9%). Age-stratified analysis revealed that *S. aureus* predominated in patients under 50, while *P. aeruginosa* was more common in those over 50 years of age. Table 2 displays details of microbial culture results.

### Medical and Surgical Treatments

A total of 222 patients (25.6%) received oral doxycycline, while 23 patients (2.5%) were treated with acyclovir. The topical antibiotic regimens are listed in Table 3.

A total of 386 patients (44.6%) required surgical treatment. Tarsorrhaphy was the most frequent procedure (23.6%), followed by tectonic graft (38.3%) and patch graft (34.8%).

Surgical intervention was pursued in cases where there was resistance to topical antibiotics, corneal perforation, or a risk to the integrity of the eye. For small perforations located in the corneal periphery, minor procedures like bandage and glue application, as well as conjunctival flap techniques, were employed. Major surgical interventions were also conducted as necessary and included tectonic grafts for central and larger corneal perforations, as well as evisceration or enucleation for endophthalmitis and cases progressing to eye loss due to corneal perforation. Some patients required multiple surgeries. Additional information about these procedures can be found in Table 4.

### Visual Outcomes 

The mean visual acuity on admission was 2.24 ± 0.66 logMAR (logarithm of the minimum angle of resolution), which improved significantly to 1.74 ± 0.96 logMAR at discharge (*P* < 0.001).

In the logistic regression model (n = 867) shown in Table 5, older age, neurotrophic keratopathy, central ulcer location, large ulcer size ( > 4 mm), corneal perforation, and prior trauma were associated with increased odds of poor final visual outcome (final VA ≥ 1.3 logMAR). Corneal perforation showed the largest effect size (OR ≈ 4.75), followed by central ulcer location (OR ≈ 3.10) and neurotrophic keratopathy (OR ≈ 2.20). Contact lens use and sex were not significant predictors after adjustment. The model demonstrated acceptable calibration (Hosmer–Lemeshow P = 0.42) and good discrimination (area under the curve = 0.81).

**Table 3:** Antibacterial treatment regimens administered for patients in this research

Topical antibiotics (%)	
Ceftazidime + Vancomycin	428 (49.3)
Fluoroquinolone	138 (15.9)
Amikacin + Gentamicin	33 (3.8)
Vancomycin	21 (2.42)
Cefazolin + Gentamicin	112 (12.9)
Amikacin + Ceftazidime	12 (1.38)
Ceftazidime + Gentamicin	15 (1.73)
None	108 (12.4)
Oral medications (%)	
Doxycycline	222 (25.6)
Acyclovir	23 (2.5)
Intravenous antibiotics	136 (15.74)

**Table 4:** Antibacterial treatment regimens administered for patients in this research

Surgery (%)	
Tectonic graft	149 (38.3)
Patch graft	135 (34.8)
Conjunctival flap	105 (27.1)
AMT ${}^{*}$	18 (4.6)
Glue + BCL ${}^{**}$	24 (6.2)
Evisceration	45 (11.6)
Enucleation	4 (1.2)
No intervention (%)	-55.4
${}^{*}$ Amniotic membrane transplantation; ${}^{**}$ Bandage contact lens.	

**Table 5:** Logistic regression analysis of risk factors for poor visual outcome

**Predictor**	** β (log-OR)**	SE*	OR**	95% CI*** for OR	***P*** **-value**
Age (per decade)	0.223	0.07	1.25	1.09 to 1.43	0.0014
Sex	0.049	0.085	1.05	0.89 to 1.24	0.56
Trauma	0.616	0.22	1.85	1.22 to 2.81	0.0049
Contact lens	0.095	0.12	1.1	0.86 to 1.36	0.43
Blepharitis	0.3	0.18	1.35	0.94 to 1.94	0.095
Neurotrophic keratopathy	0.788	0.2	2.2	1.44 to 3.36	< 0.001
Central ulcer location	1.131	0.18	3.1	2.09 to 4.61	< 0.001
Large ulcer size ( > 4 mm)	0.693	0.22	2	1.29 to 3.08	0.0016
Corneal perforation	1.558	0.22	4.75	3.09 to 7.31	< 0.001
Hypopyon	0.438	0.223	1.55	1.00 to 2.40	0.0495
${}^{*}$ Spherical equivalent; ${}^{**}$ Odds ratio; ${}^{***}$ Confidence interval.					

**Table 6:** Primary corneal zones, main predisposing factors, and predominant bacteria implicated in bacterial corneal ulcers by geographical region

**Study**	**Corneal zone**	**Main predisposing factor**	**Main bacteria**	**Country**
Present study	Central	Trauma	*Pseudomonas*	Iran
Ravinder et al (2016)^[11]^	Central	Trauma	*Staphylococci*	India
Ibrahim et al (2009)^[12]^	Mid-peripheral	CL wearing ${}^{*}$	*Staphylococci*	England
Mah-Sadorra et al (2005)^[13]^	Peripheral	CL wearing ${}^{*}$	Gram-positive	England
Mah-Sadorra et al (2005)^[13]^	Central	CL wearing ${}^{*}$	Gram-negative	England
${}^{*}$ Contact lens wearing.				

##  DISCUSSION

Corneal opacities caused by microbial keratitis continue to represent one of the leading causes of blindness worldwide. The condition is particularly common in developing countries, with incidence estimates suggesting rates up to 10 times higher than in high-income nations.^[8]^ Unlike cataracts, which primarily affect older individuals, microbial keratitis disproportionately impacts people in their younger, productive years, creating long-term socioeconomic consequences.^[8]^

The financial burden is also considerable. Costs are linked not only to direct medical treatment but also to visual impairment, the need for corneal transplantation, and the overall impact on quality of life.^[9]^ In the USA, the average cost of medication for bacterial keratitis is $933 per patient, while in the UK it is estimated at £ 2855.^[9]^ Although comparable data from low-income countries are lacking, the impact is likely more severe due to delayed presentation, limited healthcare infrastructure, and higher rates of untreated vision loss.^[9]^ Consequently, the disease disproportionately affects poorer populations at the most productive stages of life.^[9]^

Predisposing factors for corneal infection vary between industrialized and low-income countries. In high-income regions, contact lens wear accounts for 35-65% of cases with bacterial keratitis, followed by ocular trauma, previous eye surgery, and ocular surface disease.^[5,9,10]^ In contrast, trauma is the leading cause in low-resource settings.^[9]^ Our study confirmed trauma as the main risk factor, consistent with previous research in Iran^[1]^ and India.^[11]^ By contrast, studies in Australia^[2]^ and the UK^[12,13]^ identified contact lens wear and corticosteroid use as the predominant contributors.

Our findings showed that the central cornea was most frequently affected, followed by mid-peripheral and peripheral areas. Ibrahim et al identified contact lens use as the main cause of bacterial corneal ulcers in the UK, with predominating and ulcer location differing between lens wearers and non-wearers.^[12]^ Mah-Sadorra et al further noted that Gram-positive ulcers typically affect the peripheral cornea, while Gram-negative ulcers involve the central cornea.^[13]^ These findings are summarized in Table 6.

Central involvement is known to be associated with poorer outcomes due to its higher risk of perforation and significant visual impairment.^[14]^ In our cohort, 14% of patients developed perforated ulcers, a rate substantially higher than the 2.8% reported in England^[12]^ but comparable to the 17% observed in Oman, where *Pseudomonas* was also a dominant pathogen.^[15]^ These differences highlight the influence of regional risk factors, particularly trauma involving organic material, which was strongly associated with corneal perforation in our study.^[16]^

In the present study, regression analysis confirmed central ulcer location, corneal perforation, and older age as independent predictors of poor visual outcome. This is in line with previous reports from India^[13]^ and Oman,^[15]^ where central ulcers and perforation were strongly correlated with worse vision and higher rates of surgical intervention.^[13,15]^ Central corneal ulcers directly threaten the visual axis and often require tectonic grafts. Corneal perforation was the strongest predictor in our cohort, highlighting the destructive potential of advanced infection when management is delayed. Older age has also been recognized as a risk factor, likely reflecting ocular surface changes, immune decline, and slower healing responses. By contrast, factors such as contact lens wear and sex were not significant in our model, which may reflect regional patterns of exposure and health-seeking behavior. Collectively, these results emphasize the need for early recognition and aggressive treatment in elderly patients and in cases with central ulcers or thinning to prevent irreversible vision loss.

In 57.6% of our cases, no bacterial growth was detected. This high rate of negative cultures may be explained by factors such as sampling errors, the use of bacteriostatic anesthetics, or prior antibiotic administration before corneal scraping. Similar challenges have been reported in other retrospective studies. Among culture-positive cases, *P. aeruginosa* (28.2%) and *S. aureus* (23.9%) were the predominant isolates. These findings are consistent with previous studies in Iran^[7]^ as well as reports from Italy,^[17]^ Bahrain,^[18]^ Oman,^[14]^ Malaysia,^[19]^ Taiwan,^[20,21]^ Thailand,^[22]^ Singapore,^[22]^ and the Philippines,^[22]^ all of which identified *Pseudomonas* as the leading cause of bacterial keratitis. By contrast, studies from the USA,^[5]^ UK,^[23]^ Switzerland,^[24]^ France,^[25]^ Spain,^[26]^ Japan,^[27]^ South Korea,^[28]^ Brazil,^[29]^ and India^[30,31]^ more often reported Gram-positive bacteria such as coagulase-negative *staphylococci*, *S. aureus*, or *S. pneumoniae* as the major pathogens.

Geographical variation likely reflects differences in climate, socioeconomic conditions, occupational exposures, and common predisposing factors.^[9]^ Infections following ocular surgery or pre-existing eye disease are often caused by Gram-positive bacteria, whereas keratitis resulting from trauma or contact lens wear is more frequently linked to environmental pathogens such as *P. aeruginosa*.^[9]^

Age-related patterns were also observed in our study, with *S. aureus* being predominant among patients under 50 years old and *Pseudomonas* more common in those over 50 years of age. This may be partly explained by the growing popularity of refractive surgeries in younger individuals, which may increase the risk of *staphylococcal* keratitis.^[32]^ In older patients, immune decline, ocular surface changes such as dry eye disease, and higher rates of comorbid conditions may increase susceptibility to *Pseudomonas*.^[33,34]^ The tear film and immunoglobulins normally serve as protective barriers, but their effectiveness declines with age.^[33]^ Additionally, contaminated lubricants and bandage lenses, often used in elderly patients with ocular surface disease, can serve as reservoirs for *Pseudomonas* infection.^[35]^ These findings differ from the results of Soleimani et al's study,^[7]^ who reported *Pseudomonas* as the predominant microorganism in patients under 50 years old and *S. pneumoniae* in those over this age. They attributed this to the popularity of cosmetic contact lenses among younger patients and the higher prevalence of nasolacrimal duct obstruction among older individuals.^[7]^ Such contrasting results underscore the importance of local epidemiological surveillance in guiding empirical therapy.

The cornerstone of bacterial keratitis management is early initiation of topical antibiotics.^[3]^ Fluoroquinolones, particularly ciprofloxacin and levofloxacin, are widely used as first-line agents due to their broad coverage, excellent ocular penetration, and minimal toxicity.^[36,37]^ However, growing fluoroquinolone resistance in our region^[7]^ necessitated the use of fortified antibiotics such as vancomycin and ceftazidime in many of our cases. Although *Pseudomonas* infections often respond well to effective antibiotics, resistance is increasingly observed and associated with poor outcomes.^[38]^

Despite aggressive medical therapy, nearly half of our patients (44.6%) required surgical intervention. Tarsorrhaphy was performed in 23.6% of cases during hospitalization, while more advanced procedures were required for nonresponsive or severe ulcers. Small peripheral perforations were managed with cyanoacrylate glue and bandage contact lenses or conjunctival flaps, while larger peripheral perforations required corneal patch grafts. Also, central or large perforations necessitated tectonic corneal grafts to restore globe integrity and preserve the visual axis.

Adjunctive surgical approaches such as conjunctival pedicle grafts and AMT were also employed. These not only maintain structural integrity but also offer anti-inflammatory and healing properties, thereby reducing scarring.^[39]^ In advanced cases complicated by endophthalmitis or irreversible damage, evisceration or enucleation was unavoidable. Globally, microbial keratitis accounts for up to 20% of all corneal grafts and is the leading indication in nearly one-quarter of countries.^[40]^ Success rates vary by etiology, with bacterial keratitis demonstrating the highest anatomical survival rate compared with fungal or mixed infections.^[39,40]^ This tiered approach highlights the critical role of surgery in the management of bacterial keratitis, particularly in severe, central, or refractory cases. Early recognition of the need for surgical intervention can prevent irreversible vision loss or loss of the eye.

This large, retrospective study provides valuable insights into bacterial keratitis in Iran over a 12-year period. The data identified trauma as the leading risk factor and *P. aeruginosa* as the most common pathogen, emphasizing the importance of local epidemiology for guiding empirical treatment strategies. Additionally, the findings contribute to preventive measures by highlighting modifiable risk factors such as ocular trauma. Nevertheless, the study has limitations inherent to its retrospective design. Not all patients underwent microbiological assessment, sensitivity testing was not performed; and detailed classification of trauma or ulcer morphology (e.g., infiltrate pattern and satellite lesions) was not possible. The role of nasolacrimal duct obstruction, relevant in *Pneumococcal* keratitis, was not evaluated either. Furthermore, referral bias likely influenced the findings, as many patients were already receiving treatment before presentation.

Despite these limitations, the large sample size and long study period strengthen the reliability of the results. Future prospective studies with standardized microbiological testing, antibiotic sensitivity profiling, and comprehensive risk factor documentation are essential for refining prevention and treatment strategies.

In summary, corneal trauma was the primary risk factor for bacterial keratitis in our tertiary referral center. *P. aeruginosa* was the most prevalent pathogen, with *S. aureus* more common among younger patients. These findings provide region-specific insights for empirical therapy and prevention strategies.

##  Financial Support and Sponsorship

None.

##  Conflicts of Interest

None.
